# Supplementary material for: Predicting visual function by interpreting a neuronal wiring diagram
Source: Nature. 2024 Oct 2;634(8032):113–23. doi: 10.1038/s41586-024-07953-5 (PMC11446822; doi:10.1038/s41586-024-07953-5)
Supplement: Supplementary file 6 — CRF and ERF predictions for individual TmY4 and TmY9 cells. Analogous to Supplementary Data 3, but for TmY target types. Shown are the top four monosynaptic pathways, the strongest pathway passing through each of the top ten intermediary types (ranking from Extended Data Fig. 7), and the trisynaptic pathway Tm1–TmY–Dm3–TmY (see the section entitled Prediction of spatial normalization). [file 41586_2024_7953_MOESM6_ESM.zip › DataS4/TmY9qΓèÑ/720575940628819561.pdf]

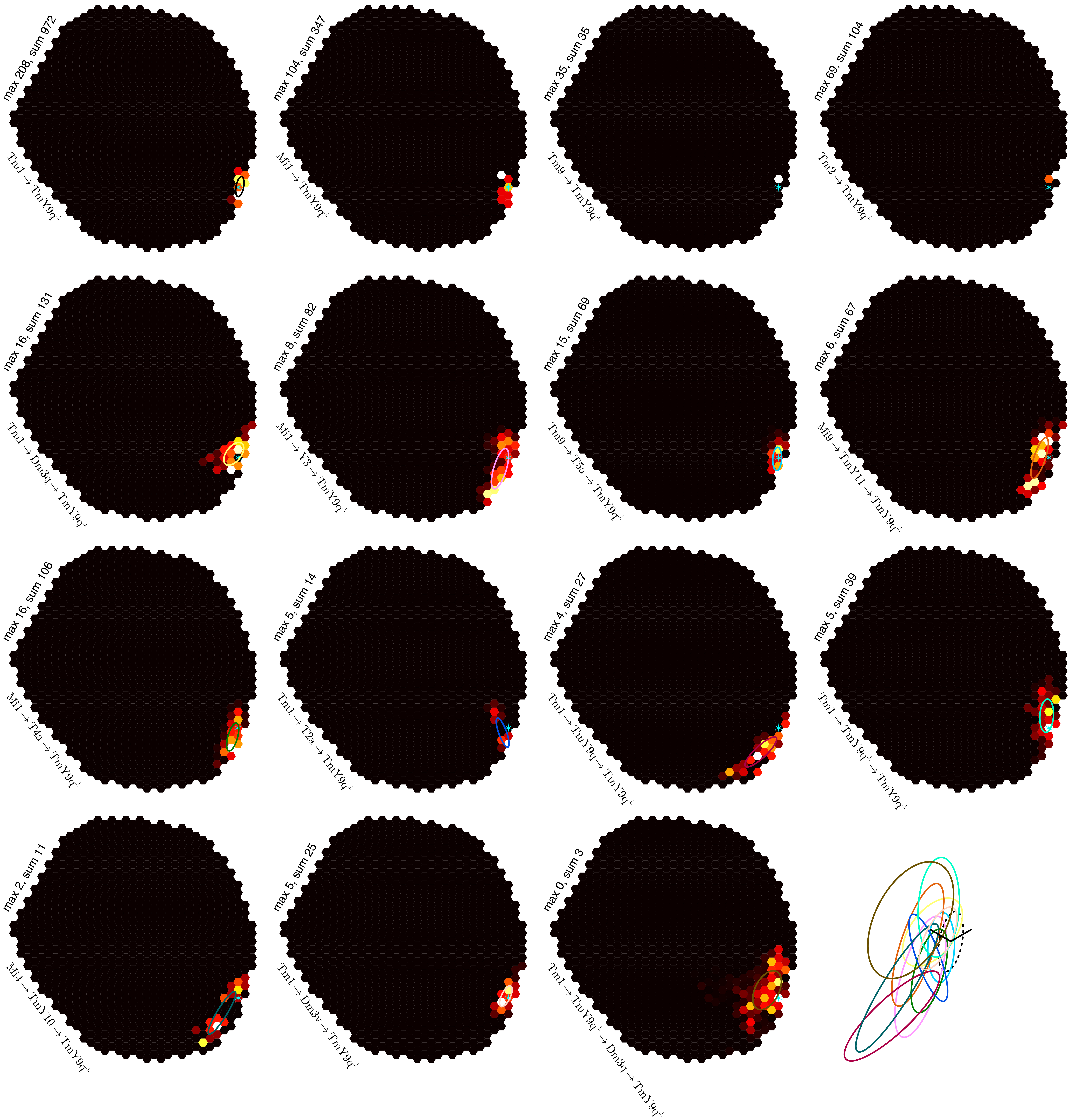
max 208, sum 972
Tm1 → TmY9q$^\perp$
max 104, sum 347
Mi1 → TmY9q$^\perp$
max 35, sum 35
Tm9 → TmY9q$^\perp$
max 69, sum 104
Tm2 → TmY9q$^\perp$
max 16, sum 131
Tm1 → Dm3q → TmY9q$^\perp$
max 8, sum 82
Mi1 → Y3 → TmY9q$^\perp$
max 15, sum 69
Tm9 → T5a → TmY9q$^\perp$
max 6, sum 67
Mi9 → TmY11 → TmY9q$^\perp$
max 16, sum 106
Mi1 → T4a → TmY9q$^\perp$
max 5, sum 14
Tm1 → T2a → TmY9q$^\perp$
max 4, sum 27
Tm1 → TmY9q → TmY9q$^\perp$
max 5, sum 39
Tm1 → TmY9q$^\perp$ → TmY9q$^\perp$
max 2, sum 11
Mi4 → TmY10 → TmY9q$^\perp$
max 5, sum 25
Tm1 → Dm3v → TmY9q$^\perp$
max 0, sum 3
Tm1 → TmY9q$^\perp$ → Dm3q → TmY9q$^\perp$
